# Patterns of utilization and determinants of maternal health services among women residing in low-income communities in Lagos State, Nigeria

**DOI:** 10.1371/journal.pgph.0004862

**Published:** 2025-09-24

**Authors:** Tope Olubodun, Onikepe Owolabi, Oluseun Adejugbe, Olufunke Iroko, Chiamaka Uwalaka, Bosede Afolabi

**Affiliations:** 1 Maternal and Reproductive Health Research Collective, Lagos, Lagos, Nigeria; 2 Department of Community Medicine and Primary Care, Federal Medical Centre Abeokuta, Abeokuta, Ogun, Nigeria; 3 Guttmacher Institute, New York, New York, United States of America; Jhpiego, UNITED STATES OF AMERICA

## Abstract

Maternal and perinatal morbidity and mortality can be significantly prevented when women utilize health facilities for antenatal care (ANC), delivery and postnatal care; particularly in low-income communities in sub-Saharan Africa, where facility-based maternal care is underutilized. This study assessed the pattern of utilization and determinants of uptake of maternal health services among women residing in low-income communities in Lagos State, Nigeria. This was a mixed methods cross-sectional study, among women of reproductive age 15 – 49 years. Quantitative data was collected from 3,651 women using interviewer-administered questionnaires. Twenty Focus Group Discussions were carried out among 172 women. Univariate, bivariate and multinomial regression was done to identify determinants of place of delivery.The mean age of respondents was 32 ± 6.4 years. Almost all the women (97.7%) had ANC during their last pregnancy regardless of provider. During ANC visits, 56.4% were attended to by a nurse/midwife, 24.2% by a doctor and 19.4% by a traditional birth attendant (TBA). Thirty-nine per cent of respondents had their last child delivered in a public health facility, 30.8% in a private health facility, and 30.2% at a TBA/religious centre/home. Determinants of facility delivery utilization include higher levels of education, higher household incomes, middle and rich wealth index, fewer number of children and higher level of satisfaction with healthcare facilities.. Reasons for choice of place of ANC and delivery from the qualitative inquiry included distance from homes, attitude of health workers and quality of care, affordability, choice of spouse, and belief in herbs/spiritual beliefs.A significant proportion of women delivered with TBA/religious centre/at home. To improve use of health facilities for maternal care, efforts must be steered at improving health worker attitudes, addressing geographical accessibility and affordability, promoting health insurance, and carrying along all relevant stakeholders including spouses, and religious and traditional leaders.

## Introduction

Each day, about 800 women die from preventable causes related to maternal and childbirth, with one woman dying every two minutes [1]. Even though maternal mortality rates dropped by about 34% between 2000 and 2020, many countries are far from achieving the sustainable development goals maternal mortality ratio (MMR) target of 70 per 100,000 live births. Ninety-five percent of maternal deaths occur in low- and middle-income countries, and Africa accounts for 69% of maternal deaths, with an MMR of 531 per 100,000 live births [2], compared with an MMR of 19.3 per 100,000 live births in the United States of America [3] and 13.41 per 100,000 live births in the UK [4] South Sudan, Chad and Nigeria have the highest MMRs in Africa. Nigeria alone was reported to have 82,000 maternal deaths in 2020, representing 28.5% of global maternal deaths [5]. The global burden of perinatal mortality is also huge, consisting of 47% of all Under-5 deaths [6]. There were around 2.3 million neonatal deaths globally in 2022, and sub-Saharan Africa had the highest neonatal mortality rate (NMR) at 27 deaths per 1000 live births [6]. Nigeria’s perinatal mortality rate (PMR) is high, at 40.9 per 1,000 births [7] which is higher than the pooled estimate for West Africa which is 35.7 per 1,000 births [8].

The leading causes of maternal mortality in Nigeria are haemorrhage, pre-eclampsia or eclampsia, sepsis, ruptured uterus, complications of unsafe abortions and prolonged obstructed labour [9,10]. The major causes of perinatal mortality in Nigeria are sepsis, intrapartum injury, low birth weight, pneumonia, jaundice and meningitis [11,12]. The majority of maternal and perinatal deaths are preventable by interventions provided during antenatal care (ANC), skilled delivery, and post-natal care (PNC) [13–15]. Access and utilization of quality maternal health services is therefore crucial to obtaining positive maternal and neonatal outcomes.

The components of ANC, which include health education and health promotion, risk identification, and prevention and management of pregnancy-related or concurrent diseases play a key role in improving maternal health [13]. Delivery by a skilled attendant and access to emergency obstetric care is also very important as approximately 15% of expected birth will result in life-threatening complications [14]. Every woman should deliver at a health facility to ensure prompt and adequate management of complications when they arise. Postnatal care is crucial for monitoring mother and child after delivery, promoting early breastfeeding and exclusive breastfeeding, identification of danger signs, and providing postpartum family planning, all which improve the health of the mother and child [15].

Even though maternal health services are very crucial, many women do not utilize these services for various reasons. In Nigeria, only 60% of mothers attend at least four antenatal visits, 49% of women deliver at a health facility and only 24%attend a postnatal care visit [16]. Women of low income have been shown to use maternal health services even less than the general population [17–19]. [16] Although antenatal care coverage is high in Lagos State, Nigeria, and approximately 80% of women deliver in health facilities [16], it remains important to examine the utilization of maternal health services among the urban poor. This is particularly relevant given that over one million people in Lagos live in extreme poverty, with many more residing below the poverty line [20]. In addition, there is paucity of research on the utilization of maternal healthcare services among the urban poor in Lagos.

Lagos State is a highly populous state with many informal settlements and poor neighbourhoods, existing alongside affluent neighbourhoods [21]. There are a range of facilities women can choose from to seek maternal health services which include public primary health centres, public secondary and public tertiary hospitals and private hospitals [22]. Some women choose to seek care from unorthodox centres such as traditional birth attendants, quack nurses popularly called *Aunty Nurse*, religious centres and at home [22,23]. Even though home births with the aid of skilled birth attendants, is increasingly being practiced in developing countries [24,25], this is not the case in Nigeria due to the paucity of health workers and the unhygienic conditions in many homes.

Understanding the patterns of maternal healthcare utilization and its determinants among women with low resources is crucial to informing policies and strategies to address inequitable health outcomes in urban settings in Nigeria and also to truly leave no one behind. This study assesses the pattern of utilization and determinants of uptake of maternal health services among women residing in low-income communities in Lagos State, Nigeria.

## Methods

### Ethics statement

Ethical approval was obtained from the Health Research Ethics Committee, Lagos University Teaching Hospital (ID: ADM/DSCST/HREC/APP/15434). Informed consent was obtained verbally from all participants and registered on the soft copy questionnaire uploaded on Open Data Kit (ODK) [26]. Informed consent was also obtained from parents/guardians of participants under 18 years of age, verbally and registered on the soft copy questionnaire uploaded on ODK.

### Study area

Lagos State is located in southwest Nigeria. The state has the highest urban population in Nigeria, which is 27.4% of the national population [27] despite being the smallest state in the country. The state therefore has many low-income communities and over 300 slum communities [28]. Lagos has 20 Local Government Areas (LGAs) (An LGA is the smallest administrative unit of government, and each LGA is further subdivided into wards, which are the smallest political units) [27].There are four public tertiary health facilities, 30 public secondary health facilities, and 331 primary public health facilities [29]. There are 1,394 registered private health facilities in the state [30]. Maternal healthcare is provided in the majority of health facilities in the state, even though private health facilities vary in resources and standard. Traditional birth attendants and religious centres, both lacking formally trained practitioners also provide antenatal and delivery services for women and can be found in many communities in Lagos state. They undergo informal training in the form of apprenticeship with the practiced being passed down from one traditional birth attendant to another.

### Survey design and study population

This mixed-methods cross-sectional study (questionnaire survey and focus group discussions) was conducted at baseline, prior to an initiative (*Iyaloju Initiative)* to elevate the standard of care provided by maternal care providers in PHC facilities within underserved communities in Lagos. The Iyaloju initiative provided comprehensive training in Emergency Obstetric Care (EmOC) for all PHC staff and in-cooperated Respectful Maternity Care (RMC) into in-service training across 10 Local Government Areas (LGAs) in Lagos State. The Iyaloju initiative also included mobile phone-based tracking and follow-up of mothers, to enhance maternity care, alongside refining procedures for seamless referrals from primary to higher-level healthcare facilities.

The study population for this research was women aged 15–49 years, residing in low-income communities in Lagos State. Women who had delivered at least one child, or were pregnant at the time of study were eligible for the study.

A low-income community as defined in this study is a geographic area in which the majority of residents earn substantially less than the average income level of the state. These communities often experience elevated poverty rates, inadequate access to quality education and employment opportunities, insufficient infrastructure investment, and greater exposure to environmental and public health risks [31] .

### Sampling procedure

A multi-stage sampling procedure was adopted in selecting study participants. Stage 1 involved the purposive selection of 10 LGAs, based on LGAs which had a large population size and had low-income communities within them. The LGAs selected were Agege, Ajeromi-Ifelodun, Eti-osa, Ibeju-Lekki, Kosofe, Lagos mainland, Mushin, Ojo, Oshodi and Surulere. Stage 2 involved purposively identifying a low-income community within the catchment area of the high volume PHC selected for the “Iyaloju” intervention activities in that LGA. One such community was selected in each LGA. A sampling technique which was adopted from the “spin the bottle” EPI-derived technique (a technique derived by EPI Research Inc for cluster sampling in household surveys) was used in selecting the houses in stage 3 [32]. In stage 3, a bottle was spun at the centre of the community, and any house facing the tip of the bottle was the first house sampled. Subsequently, every seventh house was selected, moving in a circular pattern from inward to outward. The fourth stage involved the selection of a household from each house, which was done via simple random sampling by balloting. In the fifth stage, any woman of reproductive age who had delivered at least one child, or was pregnant, in the selected household, and who consented to participate in the study was interviewed. When there were more than one of such women in a household, the participant was chosen by balloting.

### Data collection

Data collection and participant recruitment took place between June 12^th^ 2023 and August 7^th^ 2023. Survey data was collected from 3,651 women, in household settings, as they were being recruited in their communities. For the FGDs, women were invited to a central location in the community where the discussions took place.

Survey data was collected using a structured questionnaire containing questions on sociodemographic characteristics, reproductive history, careseeking behaviour during the most recent pregnancy, place of delivery, and satisfaction with care received at facilities. Women were interviewed verbally with the fieldworker entering their responses onto a tablet computer. Fieldworkers were trained and interviews were conducted in English or the local language. All fieldworkers had previous survey experience.

Twenty FGDs were conducted among groups of eight to ten women. Discussions were guided by an interview guide to identify reasons for the choice of place for receiving maternal healthcare. Each question was followed by prompts and probes to get a deeper understanding of the subject of discussion. Each FGD was conducted by a trained research assistant. There was a note taker, and the interviews were audio recorded.

### Outcome measures

#### Received ANC during the last pregnancy.

Women were asked if they received antenatal care while they were pregnant with their last child. Responses were ‘yes’ or ‘no’.

#### Place of delivery of last child.

The women were asked the place where they delivered their last child. In the univariate analysis, responses were ‘government hospital’, ‘government health centre’, ‘government health post’, ‘private clinic’, ‘private hospital’, ‘NGO hospital’ and ‘TBA/religious centre/home’. In the bivariate and multivariate analysis, government hospital, government health centre, and government health post were recoded as ‘public facility’. Private clinic, private hospital, and NGO hospital were recoded as ‘private facility’ and TBA/religious centre/home was re-labelled as unorthodox centre.

#### Independent variables.

The independent variables used in the analysis were: age, marital status, highest level of education, religion, types of occupation, household income, wealth index, age at first childbirth, number of children, gestational age of pregnancy at ANC registration, cost of transportation to health facility, distance to health facility, and level of satisfaction with healthcare. Details on how the independent variables were computed are seen in Supplementary files (S1 Table).

### Data management

To ensure anonymity, all survey data were coded with unique identifiers and names were not collected. Similarly, transcripts were coded with unique identifiers. Audio recordings, transcripts and data from the survey were stored securely in passworded documents and passworded computers to ensure confidentiality. Only key members of the research team and the statistician had access to the documents.

### Data analysis

Data entry and cleaning was done on Microsoft Excel 365 software. Data was exported to and analysed using Stata software [33].

Our initial analysis focused on describing the sociodemographic characteristics of women, and their pattern of healthcare utilization in their most recent pregnancy using frequency tables. Thereafter factors associated with the use of government facilities, private facilities or traditional birth attendants for delivery were investigated using chi2 tests. In addition, we used multinomial logistic regression to investigate the determinants of place of delivery. All quantitative analyses were conducted using Stata software [33].

For the FGDs, all audio-taped sessions were transcribed verbatim in the language the FGD was conducted and thereafter translated into English Language where necessary. The transcriptions were handled by experts in the local language and English language. A team was also involved in checking the quality of the transcripts to preserve the meanings and interpretations, as used by the participants. All the transcripts were coded by three team members, and the analysis was done with NVivo software [34]. Braun and Clarke’s thematic approach was adopted in the analysis which includes familiarization with the data, generating initial codes, searching for themes, reviewing themes, refining and naming themes, and report writing [35].

## Results

The number of women interviewed for the quantitative data collection was 3,651 (approximately 360 women per LGA). The mean age of respondents was 32 ± 6.4 years. Most of the respondents had attained secondary education (62.0%), 78.2% were currently married, more than half (55.4%) were Christians and 82.0% had household size of 1–5. The predominant occupation was trading (54.8%). Many (28.9%) of the women had household income between $107.90 and $168.00. Only 5.7% had health insurance (Table 1).

**Table 1:** Socio-demographic characteristics of respondents.

Variables	Frequency (n = 3651)	Percentage
**Age in years**		
15–24	525	14.4
25–34	1905	52.1
35–44	1124	30.8
≥ 45	97	2.7
Mean* *± SD	32 ± 6.4	
**Marital status**		
Never in a union	112	3.1
Not currently in union: Divorced/separated	81	2.2
Not currently in union: Widow	48	1.3
Currently married	2857	78.2
Living with a man	553	15.2
**Highest level of education**		
No formal education	351	9.6
Primary	402	11.0
Secondary	2264	62.0
Tertiary	634	17.4
**Able to read sentence in English**		
Able to read only part of the sentence	1241	34.0
Able to read the whole sentence	1853	50.7
Blind/visually impaired	3	0.1
Cannot read at all	554	15.2
**Religion**		
Christianity	2023	55.4
Islam	1613	44.2
Traditionalist	15	0.4
**Types of occupation**		
Unemployed	306	8.4
Agriculture	9	0.2
Blue collar job	1102	30.2
Trading	2000	54.8
White collar job	234	6.4
**Household income (monthly)**		
Below ₦30,000 (Below $46.2)	312	8.6
₦30,000 – ₦69,999 ($46.2- $107.8)	622	17.0
₦70,000 – ₦109,99 ($107.9- $169.49)	1030	28.2
₦110,000 – ₦149,99 ($169.50-$231.09)	384	10.5
₦150,000 – ₦189.99 ($231.10-$292.79)	673	18.4
≥ ₦190,000 (≥$292.80)	630	17.3
**Wealth index**		
Poor	1219	33.4
Middle	1217	33.3
Rich	1215	33.3
**Has health insurance**		
No	3444	94.3
Yes	207	5.7
**Provider of health insurance (n = 207)**		
NHIS	55	26.6
Others	4	1.9
Private	22	10.6
Provided by another member of the family	65	31.4
Provided by work	61	29.5

NHIS – National Health Insurance Scheme Dollar exchange rate Aug 2022: $1 = N649.

Eighty-four percent of women had given birth and 15.8% were currently pregnant. Most women (44.5%) had their first childbirth between the ages of 20 – 24 years and the majority (94.8%) had 1 – 4 children (Table 2).

**Table 2:** Reproductive health characteristics of respondents.

Variables	Frequency (n = 3651)	Percentage
**Ever given birth**		
Yes	3074	84.2
Currently pregnant	577	15.8
**Age at first childbirth (in years) (n = 3074)**		
< 20	374	12.2
20–24	1369	44.5
25–29	1012	32.9
≥ 30	319	10.4
Mean ± SD	24 ± 4.7	
**Number of children (n = 3074)**		
1–4	2915	94.8
> 4	159	5.2
**Age of the last child (in years) (n = 3074)**		
< 1	693	22.5
1–4	1886	61.4
5–9	433	14.1
≥ 10	62	2.0
Mean ± SD	2 ± 2.7	

Almost all the women (97.7%) had ANC during their last pregnancy, regardless of the place they sought ANC care. Fifty-six percent of the women were attended to by a nurse or midwife during ANC, 24.2% were attended to by a doctor, and 19.4% by a traditional birth attendant(TBA). Forty-four percent of the women had their first ANC visit after 13 weeks of gestational age. Eleven percent of women received ANC in more than one health facility and the reasons given include: for personal reasons (18.4%), because of a medical complication (18.2%), family decision (14.0%), distance (10.7%), belief in TBAs/herbs (9.4%) and spiritual reason (9.4%). Delivery was assisted by a nurse/midwife for 69.2% of women, by a doctor for 33.1%, and by a traditional birth attendant for 16.9% (Table 3).

**Table 3:** Pattern of utilization of maternal health services.

Variables	Frequency	Percentage
**Received ANC during the last pregnancy (n = 3074)**		
No	71	2.3
Yes	3003	97.7
**Gestational age of pregnancy at ANC registration (n = 3003)**		
≤ 13 weeks	1671	55.6
> 13 weeks	1332	44.4
**Health worker that attended to you during ANC (n = 3003)**		
Doctor	727	24.2
Nurse/midwife	1693	56.4
TBA	583	19.4
**Received ANC services in more than one facility (n = 3003)**		
No	2656	88.4
Yes	347	11.6
**Other facility ANC was received (n = 347)** ^m^		
Government health centre	161	46.4
TBA	149	42.9
Church/Religious centre	62	17.9
Government hospital	32	9.2
Private clinic	28	8.1
Maternity Home	24	6.9
Another home	5	1.4
Government health post	4	1.2
Own home	2	0.6
NGO hospital	1	0.3
**Reasons for receiving ANC in more than one facility (n = 347)** ^m^		
Personal reason	71	18.4
Medical Complication	70	18.2
Family decision	54	14.0
Distance	41	10.7
Spiritual reason	36	9.4
Belief in TBA/herbs	36	9.4
Lack of good equipment	32	8.3
Attitude of the staff	21	5.5
Cost	11	2.9
Change of residence	10	2.6
Referral	3	0.8
**Health worker who assisted with the delivery of last child** ^m^		
Doctor	993	33.1
Nurse/midwife	2079	69.2
Auxiliary midwife	202	6.7
Traditional birth attendant	508	16.9
No one assisted	30	1.0
**Place of delivery of last child (n = 3074)**		
Government hospital	345	11.2
Government health centre/health post	857	27.9
Private hospital/clinic	932	30.3
NGO hospital	16	0.5
TBA/religious centre/home	924	30.1
**Last child delivered by caesarean section (n = 3074)**		
No	2759	89.8
Yes	315	10.2
**Delivered in the same facility where ANC was received (n = 3074)**		
No	497	16.2
Yes	2577	83.8
**Reasons for delivery in another facility (n = 426)** ^m^		
Spouse’s decision	117	27.5
Medical complication	93	21.8
Distance	90	21.1
Poor attitude of the health providers	52	12.2
Lack of equipment and/or drugs	38	8.9
Unavailability of transport	27	6.3
Cost	18	4.2
Can’t guarantee confidentiality	10	2.4
Cost of transportation to health facility^*^ **(n = 3003)**		
< $0.77	2573	85.7
≥ 0.77	430	14.3
**Distance to the health facility (n = 3003)** ^m^		
Less than 10 minutes distance through walking or transportation	1360	45.3
Within 10–15 minutes of transportation	1126	37.5
Within 15–30 minutes of transportation	394	13.1
More than 30 minutes through transportation	123	3.9
**Level of satisfaction with healthcare**		
Low Satisfaction	831	22.7
Higher Satisfaction	2823	77.3

^m^ Multiple response allowed

* Cost of transportation to ANC facility.

Twenty-three percent of respondents had their last child delivered in a private hospital, 24.1% in a government health centre, and 30.2% in a TBA/religious centre/at home. Ten percent of respondents delivered their last child in a health facility different from where they received ANC and reasons include spouse’s decision (27.5%), medical complication (21.8%) distance (21.1%), and poor attitude of health provider (12.2%). For most of the women (84.2%), delivery was attended by one of the providers that provided ANC. Most women (45.3%) lived less than 10 minutes walking/transport distance and 37.5% lived within 10 – 15 minutes of transportation from the health facility (Table 3).

**Fig 1:**
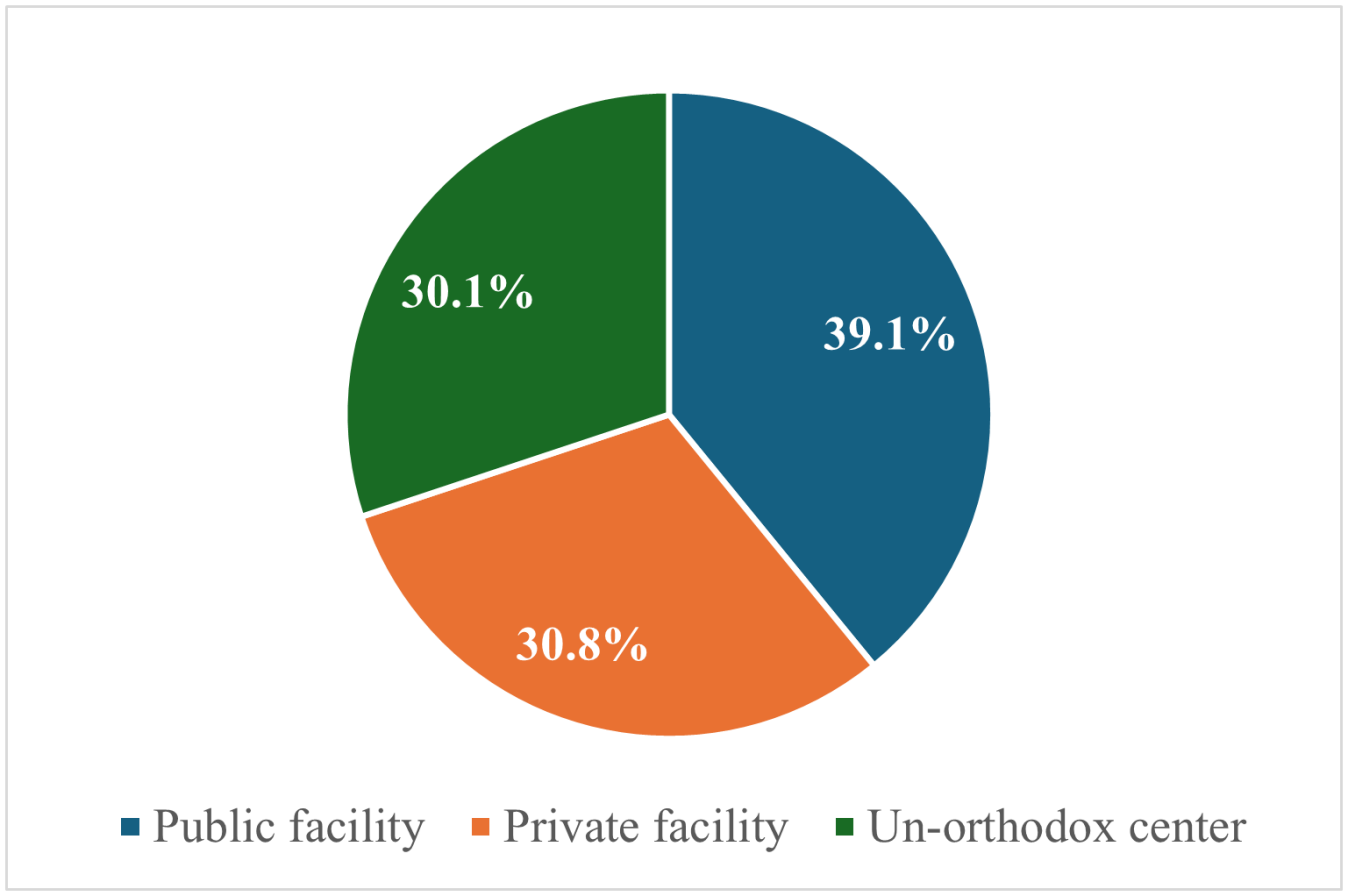
Place of delivery of the last child among respondents.

Thirty-nine percent of the women delivered their last child in a public facility, 30.8% in a private facility and 39.1% in an unorthodox centre (Fig 1).

### Age

Regarding usage of public facilities, utilization was highest among women ≥45 years (41.7%) followed closely by women aged 25 – 34 years old (41.5%). For private facilities, women aged 35–44 years had the highest utilization (36.2%). Regarding the use on unorthodox centres, younger women aged 15–24 years (40.8%), utilized unorthodox centres the most. The associations were statistically significant (p < 0.001) (Table 4).

**Table 4:** Bivariate analysis showing factors associated with place of delivery.

Variables	Public facility	Private facility	Unorthodox centre	Chi-square value	p-value
**Age in years**					
15–24	122(39.5)	61(19.7)	126(40.8)	**43.442**	**<0.001**
25–34	666(41.5)	476(29.7)	462(28.8)		
35–44	374(35.1)	385(36.2)	306(25.7)		
≥ 45	40(41.7)	26(27.1)	30(31.2)		
**Marital Status**					
Never in union	21(29.6)	20(28.2)	30(42.3)	**41.293**	**<0.001**
Not currently in union: Divorced/separated/widowed	41(32.5)	39(31.0)	46(36.5)		
Currently married	1007(40.8)	783(26.1)	679(27.5)		
Living with a man	133(32.6)	106(26.8)	169(41.4)		
**Highest level of education**					
No formal education	122(40.3	46(15.2)	135(44.6)	**170.382**	**<0.001**
Primary education	133(38.1)	70(20.1)	146(41.8)		
Secondary education	717(37.9)	594(31.4)	580(30.7)		
Tertiary education	230(43.3)	238(44.8)	63(11.9)		
**Religion**					
Christianity	737(43.1)	512(30.0)	460(26.9)	**29.723**	**<0.001**
Islam	461(34.1)	431(31.9)	459(34.0)		
Traditionalist	4(28.6)	5(35.7)	5(35.7)		
**Type of occupation**					
Unemployed	82(38.1)	58(27.0)	75(34.9)	**43.193**	**<0.001**
Agricultural	3(33.3)	2(22.2)	4(44.4)		
Blue collar job	335(37.4)	307(34.2)	255(28.4)		
Trading	709(40.1)	494(28.0)	561(31.8)		
White collar job	73(38.6)	87(46.3)	29(15.3)		
**Household income (monthly)**					
Below ₦30,000 (Below $46.2)	63(24.6)	89(34.8)	104(40.6)	**131.575**	**<0.000**
₦30,000 – ₦69,999 ($46.2- $107.8)	160(29.9)	151(28.2)	225(42.0)		
₦70,000 – ₦109,99 ($107.9- $169.49)	341(40.2)	243(28.5)	268(31.3)		
₦110,000 – ₦149,99 ($169.50-$231.09)	121(38.5)	98(31.2)	95(30.3)		
₦150,000 – ₦189.99 ($231.10-$292.79)	268(46.5)	160(27.9)	146(25.4)		
≥ ₦190,000 (≥$292.80)	249(45.9)	207(38.2)	86(15.9)		
**Wealth index**					
Poor	422(41.1)	196(19.1)	409(39.8)	**156.429**	**<0.001**
Middle	412(39.4)	325(31.1)	309(29.5)		
Rich	368(38.8)	427(42.7)	206(20.6)		
**Has health insurance**					
No	1125(39.6)	874(30.0)	912(31.4)	**44.2445**	**<0.001**
Yes	77(47.2)	74(45.4)	12(7.4)		
**Age at first childbirth (in years)**					
< 20	153(40.9)	62(16.6)	150(42.5)	**89.763**	**<0.001**
20–24	550(40.2)	380(27.8)	403(32.1)		
25–29	371(36.7)	383(37.9)	235(25.5)		
≥ 30	128(40.1)	123(38.6)	65(21.3)		
**Number of children**					
1–2	630(39.5)	543(33.4)	452(27.8)	**18.440**	**0.001**
3–4	503(40.0)	373(28.9)	414(32.1)		
≥ 5	69(43.4)	32(20.1)	58(36.5)		
**Gestational age of pregnancy at ANC registration (n = 3003)**					
≤ 13 weeks	676(40.5)	597(35.7)	398(23.9)	**48.715**	**<0.000**
> 13 weeks	526(39.5)	351(26.4)	455(34.2)		
**Cost of transportation to health facility (n = 3003)**					
< $0.77	956(37.2)	837(32.5)	780(30.3)	**65.357**	**<0.000**
≥ $0.77	246(57.2)	111(25.8)	73(17.0)		
**Distance to health facility**					
Less than 10 minutes distance through walking or transportation	582(40.5)	358(24.9)	498(34.6)	**95.618**	**<0.001**
Within 10–15 minutes of transportation	430(38.2)	360(32.0)	336(29.8)		
Within 20–30 minutes of transportation	151(38.3)	166(42.2)	77(19.5)		
More than 30 minutes through transportation	39(33.6)	64(55.2)	13(11.2)		
**Level of satisfaction with healthcare**					
Low satisfaction	93(30.8)	90(29.8)	119(39.4)	**15.759**	**<0.000**
High Satisfaction	1109(40.0)	858(31.0)	805(29.0)		

Public facility (comprises Govt hospital, Govt health centre, Govt health post); Private facility (comprises Private hospital, Private clinic, NGO clinic); Unorthodox centre (comprises TBA, religious centre, home); Dollar exchange rate Aug 2022: $1 = N649.

### Marital status

Regarding usage of public facilities, utilization was highest among women that were currently married (40.8%). For private facilities, women not currently in union had the highest utilization (31.0%). Regarding the use of unorthodox centres, women never in union (42.3%), utilized unorthodox centres the most. The associations were statistically significant (p < 0.001) (Table 4).

### Highest level of education

Regarding usage of public facilities, utilization was highest among women with tertiary education (43.3%). For private facilities, women with tertiary education also had the highest utilization (44.8%). Regarding the use of unorthodox centres however, women with no formal education (44.6%) utilized unorthodox centres the most. The associations were statistically significant (p < 0.001) (Table 4).

### Religion

Regarding usage of public facilities, utilization was highest among Muslim women (34.1%). For private facilities, women who practiced traditional religion had the highest utilization (35.7%). Regarding the use of unorthodox centres also, women that practiced traditional religion (35.7%) utilized unorthodox centres the most. The associations were statistically significant (p < 0.001) (Table 4).

### Type of occupation

Regarding usage of public facilities, utilization was highest among women that were traders (40.1%), followed by women with white collar job (38.6%). For private facilities, women with white collar jobs had the highest utilization (46.3%). Regarding the use of unorthodox centres, women in the agricultural profession (44.4%), utilized unorthodox centres the most. The associations were statistically significant (p < 0.001) (Table 4).

### Household income

Regarding usage of public facilities, utilization was highest among women from households who earned $231.10-$292.79 monthly (46.5%), followed closely by women from households who earned ≥ $292.80 monthly (45.9%). For private facilities, women from households who earned ≥ $292.80 monthly had the highest utilization (38.2%). Regarding the use of unorthodox centres, women from households who earned $46.2- $107.8 monthly (42.0%) utilized unorthodox centres the most, followed by women from households who earned below $46.2 (40.6%). The associations were statistically significant (p < 0.001) (Table 4).

### Wealth index

Regarding usage of public facilities, utilization was highest among women of poor wealth index (41.1%). For private facilities, women from households of rich wealth index had the highest utilization (42.7%). Regarding the use of unorthodox centres, women from the poor wealth index (39.8%%), utilized unorthodox centres the most. The associations were statistically significant (p < 0.001) (Table 4).

### Health insurance

Regarding usage of public facilities, utilization was highest among women who use health insurance (47.2%). For private facilities also, women who use health insurance (45.4%) had the highest utilization. Regarding the use of unorthodox centres however, women who do not use health insurance (31.4%), utilized unorthodox centres the most. The associations were statistically significant (p < 0.001) (Table 4).

### Age at first birth

Regarding usage of public facilities, utilization was highest among women who had their first childbirth at <20 years of age (40.9%). For private facilities, women who had their first child at ≥30 years of age (40.1%) had the highest utilization. Regarding the use of unorthodox centres, women who had their first childbirth at <20 years of age (42.5%), utilized unorthodox centres the most. The associations were statistically significant (p < 0.001) (Table 4).

### Number of children

Regarding usage of public facilities, utilization was highest among women who had ≥ 5 children (43.4%). For private facilities, women who had 1–2 children (33.4%) had the highest utilization. Regarding the use of unorthodox centres, women with ≥5 children (36.5%), utilized unorthodox centres the most. The associations were statistically significant (p < 0.001) (Table 4).

### Gestational age of pregnancy at ANC registration

Regarding usage of public facilities, utilization was highest among women who attended their first ANC at ≤13 weeks (40.5%). For private facilities also, women who attended their first ANC at ≤13 weeks (35.7%) had the highest utilization. Regarding the use of unorthodox centres, women who attended their first ANC at >13 weeks (34.2%), utilized unorthodox centres the most. The associations were statistically significant (p < 0.001) (Table 4).

### Cost of transportation to health facility

Regarding usage of public facilities, utilization was highest among women who spent ≥ $0.77 on transportation to and from the health facility (57.2%). For private facilities, women who spent < $0.77 on transportation to and from the health facility (32.5%) had the highest utilization. Regarding the use of unorthodox centres, women who spent < $0.77 on transportation to and from the facility (30.3%), utilized unorthodox centres the most. The associations were statistically significant (p < 0.001) (Table 4).

### Distance to health facility

Regarding usage of public facilities, utilization was highest among women whose distance to the closest health facility was < 10 minutes by walking or transportation (40.5%). For private facilities also, women whose distance to the closest health facility was more than 30 minutes by transportation (55.2%) had the highest utilization. Regarding the use of unorthodox centres, women whose distance to the closest health facility was < 10 minutes by walking or transportation (34.6%), utilized unorthodox centres the most. The associations were statistically significant (p < 0.001) (Table 4).

### Level of satisfaction with healthcare

Regarding usage of public facilities, utilization was highest among women who had high satisfaction with healthcare at health facilities (40.0%) following previous visits to health facilities. For private facilities also, women who had high satisfaction with healthcare at health facilities (31.0%) had the highest utilization. Regarding the use of unorthodox centres however, women who had low satisfaction with healthcare at health facilities (39.4%), utilized unorthodox centres the most. The associations were statistically significant (p < 0.001) (Table 4).

Determinants of utilization of a public facility for delivery were level of education, religion, household income, health insurance status, cost of transportation to health facility and level of satisfaction with care. Women with tertiary education were more likely to deliver in public facilities than those with no education (aOR 2.36, 95%CI 1.43-3.55). Muslim women had lesser odds of delivering in a public facility, compared to Christian women (aOR 0.67, 95% CI 0.55-0.81). Women with household income ≥ $292.80 were three times more likely to use a public facility for delivery compared with women with household income below $46.2 (aOR 3.33, 95% CI 2.15-5.19). Women who had health insurance coverage had 2 times higher odds of delivering in a public facility (aOR 2.69, 95% CI 1.40-5.19). Women who spent $0.77 or more on transportation to health facility were more likely to deliver in a public facility, compared with women who spent less than <$0.77 on transportation. Women with high satisfaction were more likely to use public facility (aOR 1.82, 95% CI 1.47-2.25) (Table 5).

**Table 5:** Multinomial regression showing determinants of place of delivery.

Variables	Place of Delivery	
	Public facility vs unorthodox centre	Private facility vs unorthodox centre
	aOR (95% CI)	aOR (95% CI)
**Age**		
19–25	1	1
26–34	1.21(0.87 -1.70)	1.64*(1.11-2.43)
35–44	0.92(0.61-1.39)	2.04*(1.29-3.22)
45+	0.96(0.49-1.84)	1.27(0.60-2.66)
**Marital Status**		
Never in union	1	1
Not currently in union: Divorced/separated/widowed	1.52(0.70-3.27)	1.18(0.53-2.62)
Currently married	1.48(0.80-2.75)	1.13(0.58-2.17)
Living with a man	1.06(0.55-2.03)	1.06(0.53-2.10)
**Highest level of education**		
No formal education	1	1
Primary education	0.92(0.63-1.33)	1.08(0.68-1.1.73)
Secondary education	1.09(0.79-1.49)	1.71**(1.15-2.55)
Tertiary education	2.36***(1.43-3.55)	3.74***(2.24-6.21)
**Religion**		
Christian	1	1
Islam	0.67***(0.55-0.81)	0.93(0.76-1.13)
Traditional	0.88(0.20-3.49)	1.69(0.42-6.79)
**Type of occupation**		
Unemployed	1	1
Blue collar job	0.35(0.06-1.97)	0.29(0.04-1.86)
Trading	0.91(0.62-1.34)	0.94(0.61-1.43)
Agriculture	0.95(0.65-1.37)	0.76(0.50-1.15)
White collar job	0.91(0.50-1.64)	1.15(0.63-2.12)
**Household income (monthly)**		
Below ₦30,000 (Below $46.2)	1	1
₦30,000 – ₦69,999 ($46.2- $107.8)	1.16(0.78-1.72)	0.84(0.57-1.24)
₦70,000 – ₦109,99 ($107.9- $169.49)	1.94***(1.33-287)	0.88(0.60-1.27)
₦110,000 – ₦149,99 ($169.50-$231.09)	1.81***(1.16-3.15)	0.86(0.56-1.35)
₦150,000 – ₦189.99 ($231.10-$292.79)	2.61***(1.73-4.67)	0.83(0.55-1.24)
≥ ₦190,000 (≥$292.80)	3.33***(2.15-5.19)	1.30(0.85-2.01)
**Wealth index**		
Poor	1	1
Middle	0.97(0.77-1.22)	1.46**(1.13-1.89)
Rich	0.91(0.70-1.18)	2.21****(1.68-2.93)
**Has health insurance**		
No	1	1
Yes	2.69**(1.40-5.19)	2.45**(1.26-4.73)
**Age at first childbirth (in years)**		
< 20	1	1
20–24	1.09(0.82-1.45)	1.50(1.06-2.13)
25–29	1.02(0.73-1.42)	1.92**(1.31-2.83)
≥ 30	1.12(0.72-1.76)	1.76**(1.07-2.89)
**Number of children**		
1–2	1	1
3–4	0.93(0.76-1.20)	0.75(0.59-0.96)*
≥ 5	1.03(0.66-1.60)	0.56(0.33-0.94)*
**Gestational age of pregnancy at ANC registration (n = 3003)**		
≤ 13 weeks	1	1
> 13 weeks	0.78(0.64-0.94)	0.55***(0.44-0.67)
**Cost of transportation to health facility**		
< $0.77	1	1
≥ $0.77	2.39***(1.78-3.20)	1.03(0.71-1.45)
**Level of satisfaction with healthcare**		
Low satisfaction	1	1
High satisfaction	1.82***(1.47-2.25)	1.67***(1.33- 2.09)

*p<0.05, **p<0.01, ***p<0.001; Dollar exchange rate Aug 2022: $1 = N649.

Determinants of utilization of private facility for delivery include age of woman, level of education, wealth index, health insurance status, older age at first birth, gestational age pregnancy was booked, and level of satisfaction with care at health facilities (Table 5).

Older women were more likely to use private facilities than unorthodox centres. Women aged 35–44 years were two times more likely to use private facilities than women aged 19–25years (aOR2.04, 95% CI 1.29-3.22). More educated women had higher odds of delivering in a private facility, than women with no formal education. For instance, women with tertiary education were three times more likely to deliver in private facilitiesthan those with no education (aOR 3.74, 95%CI 2.24-6.21). Rich women were two times more likely to deliver in a private facility compared with poor women (aOR 2.21, 95% CI 1.68-2.93). Also, women with health insurance had higher odds of using a private health facility (aOR 2.45, 95% CI 1.26-4.73) compared with women with no insurance (Table 5).

Women who delivered their first child at older ages had higher odds of delivering in a private facility rather than unorthodox centre. For instance, women whose first childbirth was between 25–29 years had ninety percent higher odds of using private facilities compared with women whose first delivery was less than 20 years old. (aOR 1.92, 95% CI 1.31-2.83). Women who registered their pregnancies after 13 weeks gestational age were less likely to deliver in a private facility (aOR 0.55, 95% CI 0.44-0.67) compared to women who booked at less than 13 weeks gestation. Women with high satisfaction were more likely to use private facility rather than unorthodox centres (aOR 1.67, 95% CI 1.33-2.09) (Table 5).

### Results from focus group discussions

One hundred and seventy-two women took part in the Focus Group Discussions. Women identified that they seek healthcare services from different facilities including hospitals, health centres, traditional birth attendants, and church missions. The choice of a health facility is determined by different factors including the closeness of the facility to their homes, the attitude of health workers and quality of care, choice of their spouse, affordability, and waiting time.

#### Affordability.

Almost all of the women interviewed stated that pricing is a key consideration when choosing where they go for their prenatal care. Many women prefer health facilities that offer free care, even when there are closer ones. Some women prefer TBA or quack nurses because services cost less and they can pay installmentally.

*“The price of hospital is much compared to Aunty Nurse (quack nurse) so I prefer to go to Aunty Nurse than hospital because of money and God always help me”. [Mother, Trader, 29years old]*

*“It was a woman that told me about Iwaya health centre. She told me that they are very good. I took one of my daughters there. On getting there, I asked them if they are taking delivery, I was told that delivery is free and since I don’t have money at hand, that was the reason why I went there for delivery”. [Mother, Trader, 39years old]*

*“Reason why Aunty Nurse(quack nurse) hospital is good is that if you don’t have money to pay or buy things in government hospital, Aunty Nurse will still help attend to you because you’re in same area and you’ll pay later. But government hospital will never attend to you for free even if the person is dying, and it is not good”.[Mother, Nurse, 40 years old]*

#### Quality of care and health worker behaviour.

The interviewed women commented on how the behaviour or disposition of the health workers influenced the choice of the health facility that they visited. The women perceived the quality of care as the knowledge of the health workers about their condition, the ability of the health workers to provide appropriate treatment, the attitude of the health workers and waiting time. The ability of health workers to understand the condition of the women and provide adequate care is perceived as an important factor in determining the quality of care by the women.

*“At government hospital they will not attend to you well, but Aunty Nurse at home will serve you better. Government will not even look after you****”.*** *[Pregnant woman, Teacher, 24years old]*

*“I use PHC to give birth …to deliver. The doctor in the facility did not attend to me on time. They were just looking at me, so some pregnant women that I met there was the one giving me encouragement. Before they could attend to me, the baby head is already out, so I was not happy with the way I was attended”.* ***[****Mother, Artisan, 41years old]*

*“My first child was delivered at TBA and my second child was delivered at Aunty Nurse place in my area, while my 3rd child, when I want to give birth to him, I went to......a PHC. They didn’t respond on time., The attitude they gave me, make me come back home and the Aunty Nurse in my area help me take the delivery”. [Mother, Trader, 32years old]*

#### Distance to facility.

Participants identified the distance they must travel before getting to their facility of choice as an important factor in health service uptake. In many cases, participants mentioned that they selected the closest facility to them for their health patronage instead of a farther one that is preferred. In such cases, some participants may use a closer facility for antenatal and postpartum care and use the facility that they are comfortable with their services, for their delivery.

*“Where I registered for ANC is far to our house, I usually take 1000 Naira ($1.54) motor to the place, but when I want to give birth, it was in the night, so we have to make use of available option” [Mother, Trader, 32 years old]*

*“I don’t think it is wise for me to go too far distance oo…They pray very well in my church and the church is not far from my house. In fact, they won’t charge me”. [Pregnant woman, Hairdresser, 38 years old]*

#### Partner’s decision.

For some women, the decision of healthcare is made by their husbands, and this determines the facility used for ANC and delivery.

*“Is my husband who decided for me o. Since he is the one that will pay for everything....so anything he said ni. He is my oga (boss). (laughs)”. [Pregnant woman, Tailor, 45 years old]*

*“My husband has a brother who is a doctor and have his own private hospital. So, my husband would not want me to go to another hospital”. [Mother, Teacher 42 years old]*

#### Lack of equipment.

Some women avoid health facilities which are closer to their homes because they are poorly equipped.

*“The hospital beside my house, they don’t have many instruments there. They usually refer people to General Hospital. So, I don’t bother myself to register there”. [Mother, Trader, 26 years old]*

*“Most of the government hospital in my area don’t have enough equipment. So, I don’t bother to waste my time to go there. They don’t even do night shift. They close by 4pm. I did not register there o. (laughs)”... [Mother, Civil servant, 42 years old]*

#### Religious beliefs and beliefs in herbs.

Some women believe in the use of traditional rather than orthodox medicine and they believe intake of herbs provided by TBAs makes delivery smooth.

*“Me, I belive in elewe omo (TBA) pass hospital o. In elewe omo (TBA), they will give you agbo (herbal concoction) and when you want to deliver, it will be very easy”. [Pregnant woman, Trader, 25years old]*

*“They pray very well in our church. My first and second born were born there. so, this one is still going to be the same church o”. …. [Pregnant Mother, Trader, 42 years old]*

## Discussion

This study assessed the pattern and determinants of maternal health services utilization among women residing in low-income communities in Lagos State, Nigeria. Almost all the women had attended ANC in their last pregnancy, and many were attended to by a TBA only. About thirty percent of women delivered in an unorthodox centre. Women’s choice of place for ANC and delivery were influenced by distance to health facility, health worker behaviour, lack of equipment and drugs, affordability of services, spouse’s decision, unavailability of transportation and spiritual beliefs. Determinants of utilization of a public facility for delivery rather than unorthodox centre, were higher levels of education, having higher household income, using health insurance, spending more on cost of transportation to health facility and having high level of satisfaction with care. Determinants of utilization of private facility for delivery rather than unorthodox centre include higher levels of education, being of the middle and rich wealth index, having health insurance, being of older age at first birth, and having level of satisfaction with care at health facilities. Muslim women were less likely to use a public facility compared to Christian women and women that registered for ANC after the first trimester were less likely to use a private facility.

Even though almost all the women attended ANC, a significant proportion of them attended ANC with a TBA, either alone or in combination with ANC at a health facility. This finding is worrisome because women who have ANC at a TBA are more likely to also deliver with a TBA [36]. In a study in rural Ogun State Nigeria, eighty percent of women used TBA for ANC with some using health facilities as well [37]. ANC with TBA can be associated with a higher risk of having poor health outcomes due to failure to promptly identify or manage complications [38,39]. ANC with TBA and other informal providers like quack nurses, popularly called “*Aunty nurse”* should be discouraged.

Close to half of the women in this study had their first ANC after the first trimester. Early ANC initiation is highly recommended as it allows for more accurate estimates of date of delivery, initiation of early pregnancy interventions such as folic acid supplementation to prevent birth defects and makes for timely identification of risks and possible complications [13]. In a study in Ethiopia, more than sixty percent of women were late initiators of ANC [40], and in a study in PHCs in Southwest Nigeria, only 11% of women booked for ANC in the first trimester [41]. Poor understanding of the role of early ANC initiation may be responsible for delayed ANC initiation among women in our study. The cultural norm around not disclosing early pregnancy due to fear of evil attack, may also have contributed to many women not registering their pregnancy early. It is pivotal to educate women on the importance of early ANC initiation to maximize the benefits of ANC attendance.

In this study, about seventy percent of women delivered in a health facility. The Nigeria Demographic Health Survey 2018 (NDHS 2018) similarly reported that 75% of live births in Lagos takes place in a health facility. However, the rate of facility-based delivery in Nigeria, is much lower, as only 41% of women give birth in health facilities in the country [42]. Our findings, being higher than the national estimate for health facility delivery, may possibly be due to the availability of more health facilities in Lagos, as well as higher literacy among women in Lagos [43,44].

Even though many women in our study delivered at health facilities, close to thirty percent delivered in unorthodox centres. Similarly, in a study conducted in the Volta region in Ghana, 26% of women used TBAs [36] and in a study in rural areas in Ogun State, southwest Nigeria, 44% of women were currently using TBAs [37]. Delivery with a traditional birth attendant has higher risk for poor maternal and child outcomes due to a lack of skills to diagnose and manage birth complications when they arise, and as a result of unhygienic and dangerous traditional practices. The importance of skilled attendants at birth cannot be overemphasized in ensuring safer deliveries, and every woman should have access to this.

Findings from both the survey and FGD showed that distance to health facility and spousal/family decisions contribute to place of delivery. In Indonesia, a study reported that mothers who had better access to a health facility had greater odds of using a health facility for ANC [45]. Similarly, in a study in Angola, women living at a greater distance to a health facility were less likely to use maternal health care [46]. The importance of spousal/family involvement in maternal healthcare also cannot be ignored. Husbands and family members should be targeted by relevant health promotion/education programmes in order to promote maternal health.

The attitude of health workers can influence maternal health services utilization, as seen in both our quantitative and qualitative inquiry. In a study conducted in Ghana, women who had friendly experiences with health workers were less likely to deliver with a TBA [36]. Training and re-training of health workers on respectful maternity care, including adequate staffing to avoid work-overload is paramount for health workers to render services with respect and dignity to patients. Loss of women to unorthodox care as a result of health worker behaviour can and must be prevented.

Spiritual beliefs and preference for herbs influenced some women’s choice of place for ANC and delivery in this study. In the Volta region in Ghana, women who were of traditional religion were more likely to deliver with TBAs [36]. In studies carried out in rural communities in Nigeria, some participants felt specific maternal morbidities are best treated with traditional medicine [47,48]. Traditional beliefs that favour patronage of unorthodox centres for maternal healthcare need to be addressed. Religious and traditional leaders can be engaged, trained and mobilized to encourage use of health facilities among women.

Women with higher levels of education in this study, were more likely to use both a private and a public health facility for delivery. This may be because women with higher levels of education are more likely to know the benefits and importance of health facility delivery [49]. In this study also, women with higher household income were more likely to use a public facility. Affordability of services has been shown to influence the utilization of services [50]. It is worthy of note that the wealth index was a determinant of private facility utilization and not household income. While both household income and wealth index are markers of economic status, household income depicts the money available to the household, while wealth index is a measure of standard of living (as assessed by ownership of household items and type of housing and cooking materials). It is assumed that women who have a higher standard of living have higher expectations for care quality and may prefer to visit private health facilities where they have more private care, better health worker attitudes and shorter waiting times, as is popularly seen in private settings in Nigeria [51]. Health insurance status was also a determinant of health facility utilization, buttressing that affordability of services is crucial in addressing issues of health services utilization.

Women who had higher satisfaction with care received at health facilities were more likely to deliver their babies at health facilities. Health managers must strive to improve the quality of care at health facilities, including the availability of drugs and equipment, waiting times and health worker attitude. Similar to our study, in a community-based study carried out in Kombolcha district, Eastern Ethiopia, women who perceived the quality of care in health facilities as good/very good were more likely to use health facilities for ANC and delivery [52].

Our study found that women who paid more for transportation to the health facility were more likely to utilise a public facility. The higher cost of transportation was not a deterrent to seeking care, probably because affordability was not a problem for these women. Our findings also show that women with higher incomes also use public facility more. Paying more for transportation did not deter women earning more from using a health facility. This finding is however, contrary to that reported in other studies [53,54]. Women who had more children were less likely to use a private facility compared with women with one or two children. This finding is corroborated by other studies in Africa [55–57]. Women who have had more children often feel more ‘experienced’ in the birthing process and may choose to deliver in an unorthodox centre or at home, rather than in a health facility.

We found no significant associations between marital status, type of occupation and place of delivery. This may imply that a woman’s level of education and the amount of money available to spend (household income) and other factors, may be more important in determining her place of delivery, rather than whether she is single, married or separated. Also, what matters most is the amount of money available to be spent (household income) and the tendency to spend for a higher quality life (wealth index) rather than the type of occupation the woman is engaged in.

### Implications for policy and practice

Antenatal care and delivery at unorthodox centres is strongly discouraged, and efforts must be steered at improving health facility delivery rates in Nigeria. To address financial barriers to seeking care, there is a need for financial empowerment of women and girls. Poverty is a well-recognised social determinant of disease/health events [58], and this needs to be addressed in order to improve maternal and child health indices and ensure the best outcomes for mother and child. Policies and strategies need to be implemented to foster enrolment in health insurance schemes and the provision of free healthcare for the most vulnerable. Girl child education should be prioritized, especially for the economically disadvantaged, as education affects the utilization of both public and private facilities.

Quality of health services affects utilization. Health facilities need to be well-equipped to provide quality services. Governments should intensify efforts to ensure all health facilities, including primary health facilities are well-equipped and staffed. Health worker behaviour is also crucial in affecting women’s decision to use or not use a health facility. Health workers should receive periodic training on respectful maternity care, and the Government should provide an enabling environment for health workers, including ensuring sufficient workforce and good working conditions.

Strategies to encourage positive involvement of spouses and family members in maternal health decisions are needed, as spouses and family members play an important role in the choice of place of delivery. This can be achieved through community engagement and enlightenment schemes. Women also should be educated on the need to use health facilities, and traditional and religious beliefs which discourage health facility utilization need to be addressed. Women, traditional and religious leaders should be targeted for enlightenment programmes. Future studies should identify which interventions work best at promoting maternal healthcare utilization among women, spouses, family members, traditional and religious leaders.

### Strengths and limitations of the study

This study provides insight into the patterns of utilization and determinants of maternal healthcare services among women living in low-income communities, and these findings can be very useful for influencing policy and practice. This study however has a few limitations. The sampling procedure involved purposively selecting the LGAs. However, it was important to purposively select communities with low-income women to meet the study objectives. Also, a sample size formula was not used to derive the sample size, as the study was embedded within a maternal health programme. However, a very large sample was used which ensures validity of the study findings. To derive “weeks of pregnancy at first ANC visit”, most women could only provide information on how old their pregnancy was in “months”. This was converted to weeks by multiplying by 4. This could have led to an underestimation of women who began ANC after the first trimester. In addition, there is a possibility of recall bias of events that have occurred in the past.

## Conclusion

Most women had ANC and delivered their last child at a health facility. However, a significant proportion of women in these low-income communities still use unorthodox facilities for maternal care, due to socioeconomic, cultural and health system factors. Improving the attitudes of health workers, enhancing affordability through health insurance schemes, and addressing geographical accessibility are important for increasing facility-based deliveries. These findings highlight the need for multifaceted and targeted public health interventions and the inclusion of community leaders and spouses to improve maternal health outcomes.

## Supporting information

S1 Table Descriptions of independent variables. (DOCX)

## References

[1] WHO. Maternal mortality. 2023 [Accessed 2024 April 23]. https://www.who.int/news-room/fact-sheets/detail/maternal-mortality

[2] iAHO W. Analytical fact sheet maternal mortality: The urgency of a systemic and multisectoral approach in mitigating maternal deaths in Africa rationale. 2023. https://files.aho.afro.who.int/afahobckpcontainer/production/files/iAHO_Maternal_Mortality_Regional_Factsheet.pdf

[3] CDC. Provisional Maternal Death Rates. n.d. https://www.cdc.gov/nchs/nvss/vsrr/provisional-maternal-deaths-rates.htm

[4] Rimmer A. Maternal death rate in UK rises to highest level in 20 years. BMJ. 2024;384:q62. doi: 10.1136/bmj.q62 38212050

[5] Khalil A, Samara A, O’Brien P, Coutinho CM, Quintana SM, Ladhani SN. A call to action: the global failure to effectively tackle maternal mortality rates. Lancet Glob Health. 2023;11(8):e1165–7. doi: 10.1016/S2214-109X(23)00247-4 37474218

[6] WHO. Newborn mortality. n.d. https://www.who.int/news-room/fact-sheets/detail/newborn-mortality#:~:text=Overview,in%20child%20survival%20since%201990

[7] Nwokoro UU, Dahiru T, Olorukooba A, Daam CK, Waziri HS, Adebowale A, et al. Determinants of perinatal mortality in public secondary health facilities, Abuja Municipal Area Council, Federal Capital Territory, Abuja, Nigeria. Pan Afr Med J. 2020;37:114. doi: 10.11604/pamj.2020.37.114.17108 33425147 PMC7755356

[8] Akombi BJ, Renzaho AM. Perinatal mortality in sub-saharan africa: A meta-analysis of demographic and health surveys. Ann Glob Health. 2019;85(1):106. doi: 10.5334/aogh.2348 31298820 PMC6634369

[9] Sageer R, Kongnyuy E, Adebimpe WO, Omosehin O, Ogunsola EA, Sanni B. Causes and contributory factors of maternal mortality: evidence from maternal and perinatal death surveillance and response in Ogun state, Southwest Nigeria. BMC Pregnancy Childbirth. 2019;19(1):63. doi: 10.1186/s12884-019-2202-1 30744576 PMC6371466

[10] Ujah IAO, Aisien OA, Mutihir JT, Vanderjagt DJ, Glew RH. Women’s Health and Action Research Centre (WHARC) Factors Contributing to Maternal Mortality in North-Central Nigeria: A Seventeen-Year Review. 2005. 16623187

[11] Odejimi A, Quinley J, Eluwa GI, Kunnuji M, Wammanda RD, Weiss W, et al. Causes of deaths in neonates and children aged 1–59 months in Nigeria: verbal autopsy findings of 2019 Verbal and Social Autopsy study. BMC Public Health. 2022;22(1):1130. doi: 10.1186/s12889-022-13507-z 35668378 PMC9172014

[12] Abudu O, Akinkugbe A. Clinical causes and classification of perinatal mortality in Lagos. Int J Gynaecol Obstet. 1982;20(6):443–7. doi: 10.1016/0020-7292(82)90004-2 6130990

[13] WHO. WHO recommendations on antenatal care for a positive pregnancy experience. 2016. https://iris.who.int/bitstream/handle/10665/250796/9789241549912-eng.pdf?sequence=1 28079998

[14] Otolorin E, Gomez P, Currie S, Thapa K, Dao B. Essential basic and emergency obstetric and newborn care: from education and training to service delivery and quality of care. Int J Gynaecol Obstet. 2015;130 Suppl 2:S46-53. doi: 10.1016/j.ijgo.2015.03.007 26115858

[15] WHO. Raising the importance of postnatal care. n.d. https://www.who.int/activities/raising-the-importance-of-postnatal-care

[16] National Bureau of Statistics NBS, United Nations Children’s Fund UNICEF. Multiple Indicator Cluster Survey 2021, Survey Findings Report. Abuja, Nigeria: National Bureau of Statistics and United Nations Children’s Fund. 2022.

[17] Adedokun ST, Uthman OA. Women who have not utilized health service for delivery in Nigeria: who are they and where do they live?. BMC Pregnancy and Childbirth. 2019;19:1–14. 30866841 10.1186/s12884-019-2242-6 PMC6416870

[18] Ononokpono DN, Odimegwu CO, Imasiku E, Adedini S. Contextual determinants of maternal health care service utilization in Nigeria. Women Health. 2013;53(7):647–68. doi: 10.1080/03630242.2013.826319 24093448

[19] Dahiru T, Oche OM. Determinants of antenatal care, institutional delivery and postnatal care services utilization in Nigeria. Pan Afr Med J. 2015;21:321. doi: 10.11604/pamj.2015.21.321.6527 26587168 PMC4633744

[20] National Bureau of Statistics. Nigeria Multidimensional Poverty Index. 2022. https://www.nigerianstat.gov.ng/pdfuploads/NIGERIA%20MULTIDIMENSIONAL%20POVERTY%20INDEX%20SURVEY%20RESULTS%202022.pdf

[21] Olajide OA. Understanding the complexity of factors which influence livelihoods of the urban poor in Lagos’ informal settlements. 2015. https://theses.ncl.ac.uk/jspui/handle/10443/2998

[22] Oluwole EO, Akinyinka MR, Odusanya OO. Utilization of health facilities and preferred places of treatment for common health conditions in Lagos, Nigeria. Niger J Paediatr. 2019;46:15.

[23] Akinsola KO, Olasupo O, Salako J, Sanaka JZ, Samuel RN, Bakare O, et al. “I went to the primary health centre close to my workplace, but their capacity cannot deliver the baby”: exploring why women choose different providers for maternal health services in Nigeria. BMC Pregnancy Childbirth. 2025;25(1):339. doi: 10.1186/s12884-025-07382-w 40133936 PMC11934700

[24] Dunham B. Home Birth Midwifery in the United States : Evolutionary Origins and Modern Challenges. Hum Nat. 2016;27(4):471–88. doi: 10.1007/s12110-016-9266-7 27534664

[25] Johnson KC, Daviss B-A. Outcomes of planned home births with certified professional midwives: large prospective study in North America. BMJ. 2005;330(7505):1416. doi: 10.1136/bmj.330.7505.1416 15961814 PMC558373

[26] ODK. Open Data Kit. Seattle: ODK. 2024. https://getodk.org/

[27] Lagos Global. Lagos State From the Beginning. n.d. https://lagossdgandinvestment.com/tourism

[28] Lagos State Ministry of Health. List of Urban Slums in Lagos, Nigeria. 2022.

[29] Lagos State Ministry of Health. List of Public Health Facilities in Lagos, Nigeria. 2022.

[30] HEFAMAA. List of Registered Private Health Facilities in Lagos State. 2023.

[31] World Health Organization. Unmasking and overcoming health inequities in urban settings. 2010

[32] Bostoen K, Chalabi Z. Optimization of household survey sampling without sample frames. Int J Epidemiol. 2006;35(3):751–5. doi: 10.1093/ije/dyl019 16481364

[33] StataCorp. Stata Statistical Software: Release 17. College Station (TX): StataCorp LLC. 2021.

[34] QSR International Pty Ltd. NVivo qualitative data analysis software. Melbourne, Australia: QSR International. 2017.

[35] Braun V, Clarke V. Using thematic analysis in psychology. Qual Res Psychol. 2006;3:77–101.

[36] Agboyo G, Asamoah A, Ganle J, Kumah A. Factors Associated with Use of Traditional Birth Attendants for Child Delivery: A Cross-Sectional Study. Glob J Qual Saf Healthc. 2024;7(2):42–9. doi: 10.36401/JQSH-23-27 38725882 PMC11077521

[37] Ebuehi OM, Akintujoye I. Perception and utilization of traditional birth attendants by pregnant women attending primary health care clinics in a rural Local Government Area in Ogun State, Nigeria. Int J Womens Health. 2012;4:25–34. doi: 10.2147/IJWH.S23173 22371657 PMC3282603

[38] Keri L, Kaye D, Sibylle K. Referral practices and perceived barriers to timely obstetric care among Ugandan traditional birth attendants (TBA). 2010. PMC2895798 20811529

[39] Buowari OY. Traditional birth attendants issue: a menace in developing countries. Niger J Med. 2012;:466–8. 23304960

[40] Gelassa FR, Tafasa SM, Kumera D. Determinants of early antenatal care booking among pregnant mothers attending antenatal care at public health facilities in the Nole Kaba district, western Ethiopia: unmatched case-control study. BMJ Open. 2023;13(10):e073228. doi: 10.1136/bmjopen-2023-073228 37879687 PMC10603512

[41] Olufemi Olayinka T, Sebutu Bello I, Oluwafemi Olajubu T, Oloyede Oyegbade O, Omobolanle Olajubu A, Tamunotonye Ezeoma I. Factors Influencing the Booking Gestational Age Among Antenatal Clinic Attendees at Primary Health Centers in South West, Nigeria: A Cross-Sectional Study. SAGE Open Nurs. 2022;8:23779608221139078. doi: 10.1177/23779608221139078 36437894 PMC9693778

[42] National Population Commission (NPC), ICF. Nigeria Demographic and Health Survey 2018. Abuja, Nigeria, and Rockville, Maryland, USA: NPC and ICF. 2019.

[43] Makinde OA, Sule A, Ayankogbe O, Boone D. Distribution of health facilities in Nigeria: Implications and options for Universal Health Coverage. Int J Health Plann Manage. 2018;33(4):e1179–92. doi: 10.1002/hpm.2603 30091473

[44] UNICEF. Literacy among young women. n.d. https://www.unicef.org/nigeria/media/1631/file

[45] Aji RS, Efendi F, Kurnia ID, Tonapa SI, Chan C-M. Determinants of maternal healthcare service utilisation among Indonesian mothers: A population-based study. F1000Res. 2021;10:1124. 35602669 10.12688/f1000research.73847.1 PMC9086521

[46] Rosário EVN, Gomes MC, Brito M, Costa D. Determinants of maternal health care and birth outcome in the Dande Health and Demographic Surveillance System area, Angola. PLoS One. 2019;14(8):e0221280. doi: 10.1371/journal.pone.0221280 31437180 PMC6706050

[47] Ntoimo LFC, Okonofua FE, Ekwo C, Solanke TO, Igboin B, Imongan W, et al. Why women utilize traditional rather than skilled birth attendants for maternity care in rural Nigeria: Implications for policies and programs. Midwifery. 2022;104:103158. doi: 10.1016/j.midw.2021.103158 34700126

[48] Okafor IP, Sekoni AO, Ezeiru SS, Ugboaja JO, Inem V. Orthodox versus unorthodox care: A qualitative study on where rural women seek healthcare during pregnancy and childbirth in Southwest, Nigeria. Malawi Med J. 2014;26(2):45–9. 25157317 PMC4141242

[49] Aggarwal R, Thind A. Effect of maternal education on choice of location for delivery among Indian women. 2011. 22680256

[50] Bhattacharyya S, Srivastava A, Roy R, Avan BI. Factors influencing women’s preference for health facility deliveries in Jharkhand state, India: a cross sectional analysis. BMC Pregnancy Childbirth. 2016;16:50. doi: 10.1186/s12884-016-0839-6 26951787 PMC4782569

[51] Chirdan OO, Lar LA, Afolaranmi TO, Inalegwu EO, Igoh CS, Adah GU. Client satisfaction with maternal health services comparism between public and private hospitals in Jos Nigeria. Jos Journal of Medicine. 2013;7(1).

[52] Zelalem Ayele D, Belayihun B, Teji K, Admassu Ayana D. Factors Affecting Utilization of Maternal Health Care Services in Kombolcha District, Eastern Hararghe Zone, Oromia Regional State, Eastern Ethiopia. Int Sch Res Notices. 2014;2014:917058. doi: 10.1155/2014/917058 27437510 PMC4897107

[53] Atuoye KN, Dixon J, Rishworth A, Galaa SZ, Boamah SA, Luginaah I. Can she make it? Transportation barriers to accessing maternal and child health care services in rural Ghana. BMC Health Serv Res. 2015;15:333. doi: 10.1186/s12913-015-1005-y 26290436 PMC4545969

[54] Bohren MA, Hunter EC, Munthe-Kaas HM, Souza JP, Vogel JP, Gülmezoglu AM. Facilitators and barriers to facility-based delivery in low- and middle-income countries: a qualitative evidence synthesis. Reprod Health. 2014;11(1):71. doi: 10.1186/1742-4755-11-71 25238684 PMC4247708

[55] Agaba P, Magadi M, Orton B. Predictors of health facility childbirth among unmarried and married youth in Uganda. PLoS One. 2022;17(4):e0266657. doi: 10.1371/journal.pone.0266657 35390079 PMC8989320

[56] Zegeye B, Ahinkorah BO, Idriss-Wheelr D, Oladimeji O, Olorunsaiye CZ, Yaya S. Predictors of institutional delivery service utilization among women of reproductive age in Senegal: a population-based study. Arch Public Health. 2021;79(1):5. doi: 10.1186/s13690-020-00520-0 33431061 PMC7798284

[57] Olubodun T, Balogun MR, Olowoselu OI, Emina VA, Ugwuowo UU, Ogundele OO, et al. Cervical Cancer Knowledge, Risk Factors and Screening Practices among Women Residing in Urban Slums of Lagos, Southwest, Nigeria. West Afr J Med. 2022;39(6):595–602. 35749636

[58] Leskošek V. Social determinants of health: the indicators for measuring the impact on health. Zdr Varst. 2012;51:21–32.

