# Correction: Patterns of utilization and determinants of maternal health services among women residing in low-income communities in Lagos State, Nigeria

**DOI:** 10.1371/journal.pgph.0005688

**Published:** 2025-12-22

**Authors:** Tope Olubodun, Onikepe Owolabi, Oluseun Adejugbe, Oluwafunke Iroko, Chiamaka Uwalaka, Bosede Afolabi

The fourth author’s name is spelled incorrectly. The correct name is: Oluwafunke Iroko.
