# Supplementary material for: Patterns of utilization and determinants of maternal health services among women residing in low-income communities in Lagos State, Nigeria
Source: PLOS Glob Public Health. 2025 Sep 24;5(9):e0004862. doi: 10.1371/journal.pgph.0004862 (PMC12459838; doi:10.1371/journal.pgph.0004862)
Supplement: S1 Table — (DOCX) [file pgph.0004862.s001.docx]

**S1 Table**

**Descriptions of independent variables**

| **Variable** | **Descriptions** | **Categories** |
| --- | --- | --- |
| Age in years | The women were asked about their age at their last birthday. The age was categorised into age groups. | - 15-24 - 25-34 - 35-44 - ≥45 |
| Marital status | The women were asked if they were currently married or living together with a man as if married. | - Never in a union - Not currently in a union: separated/divorced - Not currently in a union: widowed - Currently married - Living with a man’ |
| Highest level of education | Respondents were asked about the highest level of school they have attended. | - No formal education - Primary education Secondary education - Tertiary education. |
| Able to read a sentence in English | A card with a simple sentence was given to the respondent to read. Respondents were classified based on their ability to read the whole sentence, part of the sentence or inability to read the sentence at all. | - Able to read only part of a sentence - Able to read the whole sentence - Cannot read at all |
| Religion | Respondents were asked what their religion was. | - Christianity - Islam - Traditionalist |
| Type of occupation | The women were asked what type of employment they were engaged in. | - Unemployed - Agriculture - Blue-collar job (regarded as manual jobs/artisans e.g. tailors, hairdressers etc) Trading, - White-collar job (regarded as people that work in offices e.g. nurses, admin officers, teachers). |
| Household income | The women were asked for the combined income of all members of their household per month. This was converted to dollars. | - < $46.2 ($46.2 corresponnds to N30,000, the country minimum wage at the time), - $46.2- $107.8 - $107.9- $169.49 - $169.50-$231.09 - $231.10-$292.79 - ≥$292.80. |
| Wealth index | The wealth index was derived as a composite measure that reflects a household's overall standard of living.[24] It was calculated by combining various household characteristics, such as ownership of household items, the materials used for walls and floors, cooking methods, and the type of fuel used for cooking. We applied Principal Component Analysis (PCA) to derive the index.[24] Tertile cut-off was used to categorize as poor, middle and rich. | - Poor - Middle - Rich. |
| Has health insurance | The women were asked if they were enrolled in any form of health insurance. | - Yes - No |
| Age at first childbirth | The women were asked the age (in years) at which they gave birth to their first child. | - <20 - 20-24 - 25-29 - ≥30 |
| Number of children | The women were asked how many children they have. | - 1-4 - >4 |
| Gestational age of pregnancy at ANC registeration | The women were asked how many weeks pregnant they were, when they first received ANC. Many women could only give answers in months, which was converted to weeks. | - ≤13 weeks - >13 weeks |
| Place of delivery (Table 3) | The women were asked the place they delivered their last child | - Government hospital - Government health centre - Government health post - Private clinic - Private hospital - NGO hospital - TBA/religious centre/home |
| Place of delivery (bivariate and multivariate analysis) | Government hospital, government health centre, and government health post were recoded as ‘public facility’. Private clinic, private hospital, and NGO hospital were recoded as ‘private facility’ and TBA/religious centre/home was re-labelled as un-orthodox centre. | - Public facility - Private facility - Un-orthodox centre |
| Cost of transportation to health facility | The women were asked how much they paid for transportation to and from the health facility | - <$0.77 - ≥$0.77. |
| Level of satisfaction with healthcare | Level of satisfaction with healthcare was assessed by asking questions on satisfaction with the following items, following previous visit(s) to a health facility: the waiting period before a health provider attended to you today, the condition of the waiting area, the discussion you had with the health worker that attended to you today, provider’s response to your health concerns, explanations you received from providers about your health concerns, privacy from having others see your examinations, the number of days in the week that the facility is open, the hours that the facility is open, the condition of the examination rooms, the availability and condition of lavatories in the facility, the availability and condition of places where you can wash your hands in the facility, and distance between the facility and your home. The total score was computed for each woman and converted to percentage. Women who scored 75% and above were categorised as having high level of satisfaction and women who scored below 75% were categorised as having low level of satisfaction. | - High level of satisfaction - Low level of satisfaction |
